# Ploidy changes in human fungal pathogens: Going beyond sexual reproduction

**DOI:** 10.1371/journal.ppat.1010954

**Published:** 2022-12-08

**Authors:** Han Du, Qiushi Zheng, Richard J. Bennett, Guanghua Huang

**Affiliations:** 1 Shanghai Institute of Infectious Disease and Biosecurity, Department of Infectious Diseases, Shanghai Key Laboratory of Infectious Diseases and Biosafety Emergency Response, National Medical Center for Infectious Diseases, Huashan Hospital, State Key Laboratory of Genetic Engineering, School of Life Science, Fudan University, Shanghai, China; 2 Institutes of Biomedical Sciences, Fudan University, Shanghai, China; 3 College of Pharmaceutical Sciences, Southwest University, Chongqing, China; 4 Department of Molecular Microbiology and Immunology, Brown University, Providence, Rhode Island, United States of America; 5 Shanghai Engineering Research Center of Industrial Microorganisms, Fudan University, Shanghai, China; 6 Shanghai Huashen Institute of Microbes and Infections, Shanghai, China; University of Maryland, Baltimore, UNITED STATES

## Introduction

Asexual ploidy shifts, which describe changes in complete sets of chromosomes via a nonsexual reproductive cycle, are widespread in nature. For example, increased ploidy levels frequently occur in certain organs of both animals and plants [1]. In many human tumors, especially some high-grade tumors, changes in ploidy are common and have been suggested to contribute to tumorigenesis [2,3]. Human fungal pathogens are a major threat to human health and cause not only superficial diseases but also life-threatening invasive or systemic infections. A number of studies indicate that asexual ploidy variations are prevalent in these species and are an important strategy for adaptation to changing environments and stresses [4]. In this Pearl, we review how ploidy changes in human pathogenic yeasts can occur independent of mating and meiosis and discuss their potential biological impacts and evolutionary significance.

### Ploidy variation is common in pathogenic fungi

Pathogenic fungi cause infections that impact the skin, mucosal surfaces, bloodstream, and internal organs. To adapt to the stressful and changing environments of different host niches, fungi have evolved a variety of strategies including phenotypic transitions, biofilm development, virulence factors, and ploidy plasticity [5,6]. Of these strategies, ploidy changes have been frequently observed in natural or clinical isolates of many human fungal pathogens. These genetic variations contribute to phenotypic diversity and the evolution of new traits and have both short- and long-term consequences for fungal biology.

### Ploidy variation in *C*. *albicans*

*Candida albicans* and the closely related species *Candida tropicalis* were long regarded as asexual diploid organisms. *C*. *albicans* is highly heterozygous and genomic evidence suggests that it could have formed by ancestral hybridization between 2 divergent lineages [7]. It is now evident that both *C*. *albicans* and *C*. *tropicalis* are able to undergo mating to form tetraploid cells, either via conventional mating between strains harboring different mating type loci (*MTL***a**/**a** or α/α) [8,9] or via same-sex mating [10]. A phenotypic transition, namely the white-opaque switch, is involved in the regulation of mating in both species [9,11,12].

Suzuki and colleagues initially claimed the discovery of haploid and tetraploid forms of *C*. *albicans* by estimating genomic content by fluorimetry [13]. However, these haploid isolates were later found to be either diploid or non-albicans *Candida guilliermondii* [14]. Hickman and colleagues were the first to identify bona fide haploid *C*. *albicans* strains that were recovered following exposure to in vitro or in vivo stresses [14]. Similar to diploid cells, haploid *C*. *albicans* cells can undergo filamentation, white-opaque switching, and sexual mating. However, haploid cells exhibited reduced growth rates and fitness remained low even after auto-diploidization, indicating that slower growth was due to the unmasking of recessive alleles during the diploid-to-haploid transition [14]. Haploid cells also showed reduced virulence in a systemic mouse infection model. While initial haploid states were unstable and spontaneously switched to the diploid state, more stable haploid forms have since been isolated that facilitate genetic analysis of the species [15].

Interestingly, the antifungal drug fluconazole was shown to directly induce tetraploid forms of *C*. *albicans* following the formation of “trimeras” consisting of binucleate, 3-lobed cells [16]. Both haploid and tetraploid forms of *C*. *albicans* are not as stable as the diploid form, and there is therefore convergence toward the default diploid state [14,17,18].

### Ploidy variation in *C*. *auris* and *C*. *glabrata*

*Candida auris* has emerged as a multidrug-resistant fungal pathogen worldwide [19], while *Candida glabrata* is the second most frequently isolated species from invasive candidiasis behind *C*. *albicans* [20]. *C*. *glabrata* is actually more closely related to *Saccharomyces cerevisiae* than to *C*. *albicans* and other *Candida* CTG clade species [20]. Both *C*. *auris* and *C*. *glabrata* are typically haploid organisms, although *C*. *auris* strains have a highly plastic karyotype and can undergo rapid stress-induced karyotypic changes [21]. Furthermore, the Huang lab recently showed the existence of diploid forms and spontaneous haploid–diploid transitions in both *C*. *auris* and *C*. *glabrata* [22,23]. Although species closely related to *C*. *auris* exhibit a sexual life cycle (e.g., *Candida lusitaniae*), sexual reproduction has yet to be described in *C*. *auris*, which in some isolates could be the result of inactivating mutations in *STE6* encoding the **a**-pheromone transporter [24].

**Fig 1:**
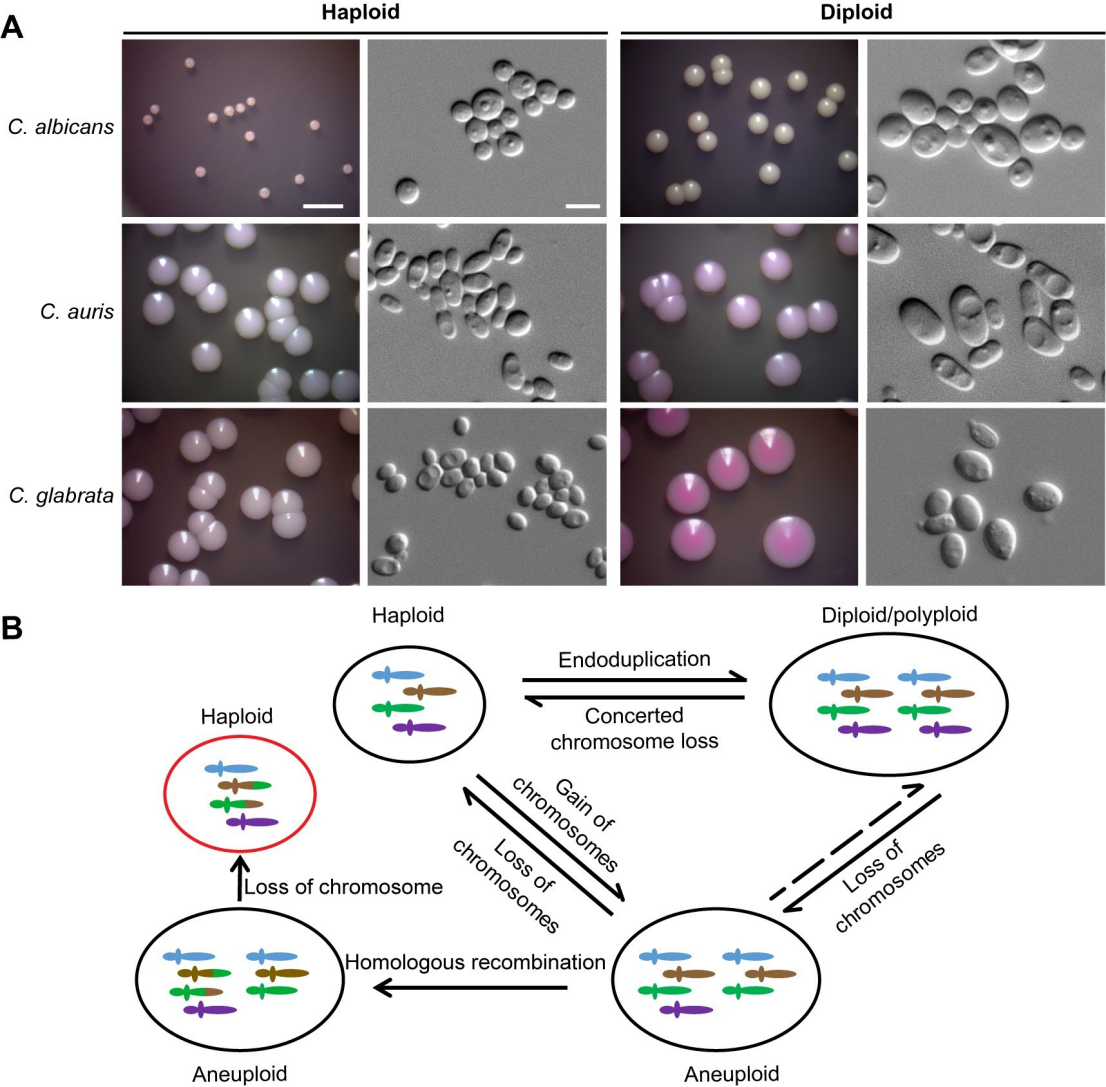
Ploidy changes in pathogenic fungi. (A) Colony and cellular morphologies of haploid and diploid forms of *C*. *albicans* (SC5314), *C*. *auris* (BJCA001), and *C*. *glabrata* (FK83). Cells were plated on YPD medium (BJCA001 and FK83 cells) or SCD medium (SC5314 cells) containing 5 μg/mL phloxine B and incubated at 30°C for 4 days. Scale bar for colonies, 5 mm; scale bar for cells, 5 μm. (B) Schematic diagram of ploidy changes. Fungal cells may undergo ploidy changes spontaneously or in response to environmental stresses. Cells of lower ploidy (e.g., haploid) can adopt a higher ploidy (diploid, tetraploid, or polyploid), which are subsequently able to return to the lower ploidy state through chromosome non-disjunction events that lead to concerted chromosome loss. Fungal cells are also able to switch between euploid and aneuploid states through gain or loss of chromosomes. Aneuploid and non-baseline ploidy states are often unstable and can give rise to additional genetic variants.

Perhaps due to technical reasons, the diploid forms of *C*. *auris* and *C*. *glabrata* natural isolates had never been observed before these reports. Experiments utilized the red dye phloxine B that differentiated colonies with different ploidies based on their color. As shown in **Fig 1**, diploid colonies of both *C*. *auris* and *C*. *glabrata* exhibited more coloration than haploid colonies [22,23]. *C*. *glabrata* haploid cells also formed darker colonies than diploid or hyperdiploid (>2C) cells when grown on CuSO_4_-containing medium [23], which could represent differences in the cellular redox state. Similar to other fungal species, diploid cells were larger than haploid cells in both *Candida* species and were also more virulent in a systemic infection model [22,23]. Using a colony staining assay together with flow cytometric analysis, the authors analyzed 500 clinical isolates of *C*. *glabrata* and showed approximately 3% of the strains were diploid or could spontaneously switch to diploid, while some isolates were even able to exist in a hyperdiploid form. Two major genetic clusters (A and B) of *C*. *glabrata* isolates were identified through multilocus sequence typing (MLST), with most isolates from China belonging to cluster A, whereas the majority of isolates from other countries belonged to cluster B. *C*. *glabrata* isolates from both genetic clusters were able to undergo ploidy changes, although isolates from cluster A demonstrated a greater haploid-to-higher-ploidy switching frequency than those from cluster B. Given the close phylogenetic relationship between *C*. *glabrata* and *S*. *cerevisiae*, a similar mechanism of endoreduplication could be involved in both species, as spontaneous diploidization events are relatively common in the latter [25].

### Polyploid titan cells in *C*. *neoformans*

The pathogenic basidiomycete *Cryptococcus neoformans* ubiquitously exists in the environment, including in the soil, on trees, and in bird droppings. *C*. *neoformans* cells are able to switch between haploid and diploid states through a sexual cycle, where the diploid state immediately undergoes meiosis and ploidy reduction. The normal cell size of *Cryptococcus* species is 4 to 10 μm and yet both *C*. *neoformans* and the closely related species *Cryptococcus gattii* are able to form “titan cells” that can reach up to 100 μm in diameter and possess a highly increased ploidy (from 4C to 312C) [26,27]. *Cryptococcus* infection begins in the lungs after spores are inhaled and approximately 20% of cells become titan cells that have a thickened cell wall and highly cross-linked capsule [26,28]. This unique cell type is thought to benefit survival and dissemination in the host due to increased resistance to environmental stresses and phagocytosis.

Interestingly, some clinical isolates of *C*. *auris* can form enlarged clusters of cells due to the failure of cell division to release daughter cells, and these may increase survival by mechanisms similar to those favoring *C*. *neoformans* titan cells. The multicellular morphology in *C*. *auris* has been demonstrated to regulate pathogenic traits and antifungal resistance [29]. Although the biological characteristics and regulatory mechanisms underlying *C*. *auris* aggregates and *C*. *neoformans* titan cells are distinct, the formation of such phenotypes may favor adaptation in both species (e.g., by enabling the avoidance of phagocytosis or due to reduced antifungal susceptibility).

## What drives ploidy changes in pathogenic fungi?

Despite the importance of ploidy changes in fungi, the mechanisms underlying such changes are poorly understood. Endoreplication/endoreduplication, where the nuclear genome is replicated in the absence of mitosis, appears to be a common stress response in eukaryotes and results in an increase in ploidy [25,30]. Studies in *C*. *neoformans* have linked ploidy increases to cell cycle regulation; one study revealed that suppression of the cell cycle regulator Cln1 can enable re-replication of DNA producing polyploid titan cells [31], while a related study showed a role for cyclins and cyclin-dependent kinases in regulating endoreplication [32]. Polyploidization can also be driven by genotoxic stresses that generate DNA double-strand breaks (DSBs) that subsequently can result in titan cell formation [33].

Recent studies have also shed new light on the fungal factors involved in ploidy reduction. In *C*. *neoformans*, the “meiosis-specific” genes *DMC1* and *REC8* were both shown to promote ploidy reduction in titan cells, which could involve a true meiosis or a para-meiotic process [33]. Parasexual ploidy reduction in *C*. *albicans* occurs in the absence of sexual sporulation yet also involves “meiosis-specific” genes, as both *SPO11* and *REC8* impacted the frequency of chromosome loss and homologous recombination [34,35]. These studies reveal a blurring of the lines between what constitutes a bona fide meiosis versus a meiosis-like process. Similar ploidy reduction processes occur in polyploid cancer cells where meiosis and recombination genes are again implicated, suggesting that related depolyploidization mechanisms occur across eukaryotes [2,3]. For example, a number of meiosis-specific genes (including DMC1, SPO11, and REC8) have been found to be activated during depolyploidization of polyploid tumor cells [36]. Changes in metabolism can also impact ploidy reduction; *C*. *albicans* tetraploid cells were unstable under conditions of high metabolic activity due to the production of reactive oxygen species (ROS) and DSBs that induce ploidy reduction [37]. Indeed, high ROS levels may have been key to DNA damage and ploidy cycling in early eukaryotes prior to the emergence of a true meiosis [37].

### Short- and long-term consequences of ploidy changes

Ploidy changes in unicellular fungi not only cause alterations in cell size, physiology, growth rate, gene expression, host adaptation, and antifungal resistance, but also can have longer-term effects on genetic changes and evolution of new traits. For example, cells of increasing ploidy often exhibit an increase in size that results in a smaller cell surface-to-volume ratio that may alter cell properties. A common feature of *C*. *albicans*, *C*. *auris*, and *C*. *glabrata* is that they are more virulent in the diploid state than in the haploid state [14,22,23]. *C*. *albicans* diploid cells are also more virulent than tetraploid cells [38], indicating that the diploid state is optimal for infections by this species.

Given the fact that sexual reproduction has not been found in many pathogenic fungi, asexual ploidy changes may represent an important alternative to sexual reproduction for the generation of genetic diversity [39,40]. Increased ploidy often leads to genome instability and could act as an intermediate state for the generation of aneuploidy, which are themselves closely linked to antifungal resistance (**Fig 1B**). Especially under stressful conditions polyploid cells generate aneuploid daughter cells at high frequency [17,18,40,41] that enables survival in harsh environments where aneuploids can be selected for and can outcompete euploid cells.

### Open questions

A number of fundamental questions remain to be addressed. For example, what underlies the different baseline ploidy states in fungi (e.g., diploid for *C*. *albicans* and haploid for *C*. *glabrata* and *C*. *auris*)? How do past hybridization events impact baseline ploidy states? Are there conserved mechanisms that control asexual ploidy changes? Did mechanisms for ploidy shifts exist before the origin of sex? How do asexual ploidy changes (e.g., random chromosome loss in high-ploidy cells) alter the generation of de novo mutations, genetic diversity, and aneuploidy? Can asexual ploidy changes serve as an alternative to sexual reproduction in “obligate” asexual species? Are there mechanisms that can increase ploidy beyond alterations to the cell cycle? And given the high frequency of fungal ploidy changes that occur in the host, what host-associated factors (or stresses) induce ploidy switching? Answers to these questions will provide greater insight into how fungi (and other eukaryotes) utilize ploidy cycling for generating phenotypic diversity and adaptation.
